# The Function of Florigen in the Vegetative-to-Reproductive Phase Transition in and around the Shoot Apical Meristem

**DOI:** 10.1093/pcp/pcae001

**Published:** 2024-01-05

**Authors:** Hiroyuki Tsuji, Moeko Sato

**Affiliations:** Bioscience and Biotechnology Center, Nagoya University, Furocho, Chikusa, Nagoya, Japan; Kihara Institute for Biological Research, Yokohama City University, Yokohama, Japan; Kihara Institute for Biological Research, Yokohama City University, Yokohama, Japan

**Keywords:** Florigen, Flowering, FT, Hd3a, SAM, Shoot apical meristem

## Abstract

Plants undergo a series of developmental phases throughout their life-cycle, each characterized by specific processes. Three critical features distinguish these phases: the arrangement of primordia (phyllotaxis), the timing of their differentiation (plastochron) and the characteristics of the lateral organs and axillary meristems. Identifying the unique molecular features of each phase, determining the molecular triggers that cause transitions and understanding the molecular mechanisms underlying these transitions are keys to gleaning a complete understanding of plant development. During the vegetative phase, the shoot apical meristem (SAM) facilitates continuous leaf and stem formation, with leaf development as the hallmark. The transition to the reproductive phase induces significant changes in these processes, driven mainly by the protein FT (FLOWERING LOCUS T) in *Arabidopsis* and proteins encoded by *FT* orthologs, which are specified as ‘florigen’. These proteins are synthesized in leaves and transported to the SAM, and act as the primary flowering signal, although its impact varies among species. Within the SAM, florigen integrates with other signals, culminating in developmental changes. This review explores the central question of how florigen induces developmental phase transition in the SAM. Future research may combine phase transition studies, potentially revealing the florigen-induced developmental phase transition in the SAM.

## Introduction

**Fig. 1:**
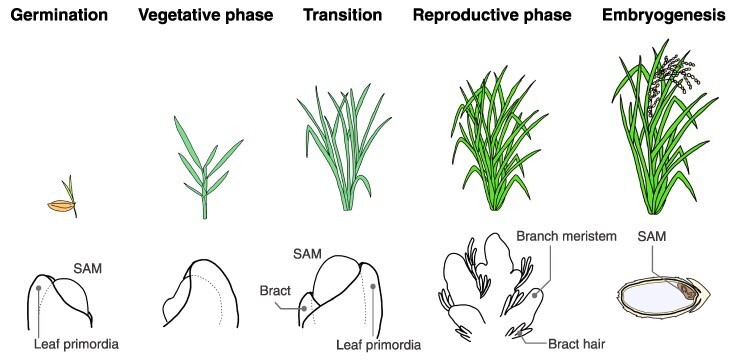
Progression of plant developmental phases. This figure provides an example using rice to illustrate the developmental phase transition. The top panel displays the overall plant morphology, while the bottom panel highlights the characteristic features of the SAMs and the surrounding tissue morphology. Germination: following germination, the SAM gives rise to the aerial parts of the plant, known as the shoot. Vegetative phase: during this phase, the SAM is responsible for leaf differentiation. Axillary meristems are formed in the leaf axils. Transition: the SAM undergoes several changes, both morphologically and physiologically. These changes include an increase in size and a shift toward the reproductive phase at the molecular level. Reproductive phase: this phase is marked by the formation of the inflorescence. Following this, the subsequent generation of SAMs arises through a series of processes: flower development, gametophyte formation and finally, fertilization. Embryogenesis: following fertilization, the SAM for the next generation is formed concurrently with seed development.

The life-cycle of plants can be conceived as a series of transitions through various developmental phases (**Fig. 1**) (Bäurle and Dean 2006, Huijser and Schmid 2011, Kyozuka et al. 2014). Each developmental phase represents a distinct developmental process, ranging from embryogenesis, seed dormancy and germination to vegetative phase characterized by leaf formation, and reproductive phase marked by flower production (Bäurle and Dean 2006, Drost et al. 2017). The life-cycle of angiosperms epitomizes this continuous progression from one developmental phase to the next. This continuum is inherently subject to the influences of the surrounding environment (Ratcliffe et al. 1999, Vicentini et al. 2023). To enhance our understanding of plant development, it is imperative to address some key questions: What are the unique characteristics that define each developmental phase? Which molecular entities force the transitions between developmental phases? In addition, how do these phase transitions arise in the context of the overall developmental process?

Previous research on the mechanism of plant development during the vegetative and reproductive phases has emphasized the central role of the shoot apical meristem (SAM) (Ratcliffe et al. 1999, Pautler et al. 2013, Tanaka et al. 2013, 2023). During the vegetative phase, the SAM drives the continuous formation of leaves and stems, including nodes and internodes, with leaf development being the hallmark of this phase. The spatiotemporal regulation of leaf differentiation and subsequent complex leaf morphogenesis establishes the plant’s form during the vegetative phase (Smith and Hake 1992). The spatial patterning of leaf primordia differentiation (known as phyllotaxis) is currently believed to be orchestrated by interactions among auxin and other plant hormones (Godin et al. 2020). The temporal interval between the leaf primordia differentiation (termed plastochron) could be regulated by the shape of the SAM and the leaf maturation rate (Smith and Hake 1992). Genes associated with enzymes and cellular signaling pathways that control the plastochron have been identified (Helliwell et al. 2001, Miyoshi et al. 2004, Kawakatsu et al. 2006, 2009, Hibara et al. 2021). In particular, the spatiotemporal organization of lateral organ primordia undergoes a dramatic transformation during the transition to reproductive phase, leading to considerable changes in phyllotaxis, plastochron and lateral organ identity (Ratcliffe et al. 1999, Kwiatkowska 2008). Significantly, in *Arabidopsis*, three distinct stages are observed based on the types of lateral organs produced. First is the vegetative phase, where basal leaves form the subtend axillary meristems. This is followed by the inflorescence 1 (I1) phase, characterized by bracts subtending outgrowing branches. The final stage is the inflorescence 2 (I2) phase, marked by the development of bractless flowers (Ratcliffe et al. 1999). These events, characterized by simultaneous changes in multiple developmental descriptors, can be referred to as a phase transition (Bäurle and Dean 2006, Kyozuka et al. 2014). What is the driving force behind such a phase transition at the molecular level? Research has highlighted the prominent role of a globular protein encoded by *Arabidopsis FT* (*FLOWERING LOCUS T*) and its orthologs, corresponding to the historical concept of ‘florigen’ (Corbesier et al. 2007, Jaeger and Wigge 2007, Lin et al. 2007, Mathieu et al. 2007, Tamaki et al. 2007, Notaguchi et al. 2008). The transition from vegetative-to-reproductive phases is daylength-dependent for many plant species. Like *Arabidopsis* and wheat, long-day plants experience accelerated flowering when daylength surpasses a genotype-determined threshold (Maeda and Nakamichi 2022, Takagi et al. 2023). Conversely, short-day plants such as rice and soybean flower earlier when the daylength falls below a specific limit (Izawa 2021, Maeda and Nakamichi 2022). For a more detailed discussion on the complex photoperiodic regulation of *FT* in phloem companion cells, readers are referred to a selection of excellent reviews that focus on this subject matter in depth (Song et al. 2015, Izawa 2021, Vicentini et al. 2023). FT and its orthologs are expressed in leaves in response to daylength, transported to the SAM and induces flowering. Hence, these proteins are long-range transducers, communicating daylength information from leaves to the SAM in plants (Tsuji 2017). The influence of these proteins on flowering varies among plant species. Rice, for example, relies entirely on *FT* orthologs for flowering and will never flower in its absence (Komiya et al. 2008). In contrast, *Arabidopsis* has alternative floral promotion pathways that can stimulate floral development even when FT is defective (Andrés and Coupland 2012). The function of mobile FT and othologous proteins and other floral promotion signals are integrated within the SAM. This integration is vital as all signals eventually induce a developmental transition in the SAM, which leads to alterations in phyllotaxis, plastochron and organ differentiation.

Considering these viewpoints, a significant question regarding vegetative-to-reproductive phase transitions resurfaces: How do the proteins encoded by *FT* or their orthologs that function as florigen induce the developmental-phase transition in the SAM? This question still needs to be solved. This review aims to encapsulate the current knowledge regarding vegetative-to-reproductive phase transitions while outlining potential future research directions. The structure of this review is as follows: first, the developmental changes that occur during the phase transition are outlined. Then, the spatio-temporal distribution of florigen in the SAM and its role in inducing these changes is discussed. This is followed by a review of the molecular processes initiated by florigen in the SAM. Finally, the implications of these discussions for research on florigen and developmental-phase transitions in the SAM are considered.

## Developmental Changes during the Vegetative-To-Reproductive Phase Transition

**Fig. 2:**
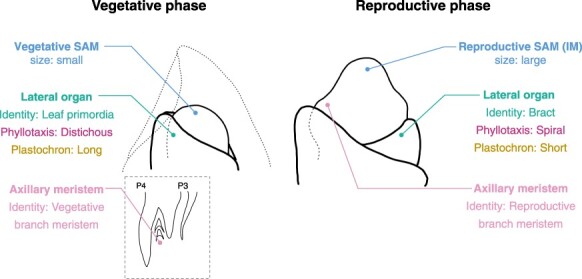
Developmental changes during the vegetative-to-reproductive phase transition in the SAM. This figure provides an example using rice to illustrate the transition. The figure presents the identity and features of the SAM, its lateral organs, and the axillary meristem. Reproductive SAM is also known as IM. Notably, the majority of these developmental characteristics undergo significant modifications during the transition.

The developmental phase transition significantly impacts nearly all developmental parameters of the SAM (**Fig. 2**) (Kwiatkowska 2008). Specifically, six distinct changes occur during this transition: SAM size and shape, phyllotaxis, plastochron, lateral organ identity, axillary meristem identity and determinacy. Notably, florigen can induce all these changes simultaneously in the SAM. While numerous comprehensive reviews are available on determinacy (Wang et al. 2018, 2023, Sakuma and Koppolu 2023, Tanaka et al. 2023), this section will focus on the other features and explore the changes that occur in these features during the developmental phase transition.

### SAM size and shape

At the onset of the developmental transition, the shape of the SAM is the most obvious morphological change observed (Kwiatkowska 2008). During this transition, the SAM enlarges and assumes a more rounded, dome-like shape. Recent and past research on *Arabidopsis* has shed light on the transition process resulting in this dome shape (Kinoshita et al. 2020). Distinct zones with diverse mitotic activities are crucial in driving these morphological changes within the SAM. The SAM consists of four primary regions: the central zone (CZ), organizing center (OC), peripheral zone (PZ) and rib meristem (RM) (Kwiatkowska 2008, Wang et al. 2018). In *Arabidopsis*, CZ is where stem cells reside and exhibit the expression of the *CLV3* gene. On the other hand, OC, which serves as the stem cell niche, is identified by the expression of the *WUS* transcription factor. PZ surrounds CZ and OC and indicates the area of differentiation for lateral organ primordia. Finally, RM, which is located at the base of the SAM, plays a vital role in growth and development. It is noteworthy that CZ and PZ are particularly affected during the developmental phase transition (Kwiatkowska 2008). As a convention, CZ displays low mitotic activity, which changes when the reproductive phase begins. During this period, there is an increase in mitotic activity within CZ. Advanced imaging studies on *Arabidopsis* revealed increased cell count and size in the SAM at the beginning of this transition (Kinoshita et al. 2020). Interestingly, gibberellic acid (GA) is essential in mediating some of these changes. A detailed analysis of reporter lines revealed dynamic changes in the expression patterns of genes involved in GA synthesis within the SAM. Although this change in SAM shape is not essential for the developmental phase transition itself in tomato (Tal et al. 2017), it is interesting to explore how it relates to controlling the timing of the developmental phase transition in other plants. As research progresses, the future objective would be to accurately identify the changes in mitotic activity and cell size in specific cells during the developmental transition, including those within the SAM.

### Phyllotaxis

Phyllotaxis plays a crucial role in creating a morphological signature that helps distinguish different developmental phases. Specific genes and their interactions orchestrate these phyllotaxis. The polarization of the PIN1 auxin transporter protein at the plasma membrane indicates the area where auxin transport is stimulated for phyllotaxis (Reinhardt et al. 2003). Polarization of PIN1 expression and localization is regulated by multiple regulatory mechanisms including PLETHORA-like AP2 domain transcription factors and the auxin-response transcription factor MONOPTEROS (Prasad et al. 2011, Bhatia et al. 2016). Phyllotaxis switching is also mediated by PLETHORA-dependent regulation of local auxin biosynthesis (Prasad et al. 2011). Rice has a significant phyllotaxis change at the reproductive phase transition (**Fig. 2**) (Tanaka et al. 2023). This transition results in changes from a 1/2 distichous pattern, where leaves are aligned in two vertical columns, to a 2/5 spiral bract arrangement. In rice, two critical genes, *ABERRANT PANICLE ORGANIZATION 1* (*APO1*) and *APO2*/*RICE FLORICAULA* (*RFL*), have been pinpointed as central players in instigating these phyllotactic changes (Ikeda et al. 2007, Ikeda-Kawakatsu et al. 2012). *APO1* in rice is an ortholog of the *Arabidopsis* gene *UNUSUAL FLORAL ORGANS* (*UFO*), which encodes the F-box protein. On the other hand, *APO2* is orthologous to the *Arabidopsis LEAFY (LFY)* gene encoding a transcription factor. Interestingly, *APO2* in rice and *LFY* in *Arabidopsis* appear to have different developmental functions with respect to floral meristem identity. In *Arabidopsis*, LFY promotes floral meristem identity acquisition (Weigel et al. 1992). LFY directly binds to key genes regulating flower development including *AP1, RAX1, ARR7, AP3, AG SEP4* and *LMI1* for floral meristem formation and activation of floral organ differentiation (Wagner et al. 1999, Moyroud et al. 2011, Winter et al. 2011, Chahtane et al. 2013). This function is shared in maize; mutations in two *LFY* orthologs in maize, *Zfl1* and *Zfl2* disrupted floral organ identity and patterning (Bomblies and Doebley 2006). In contrast, *LFY* ortholog in rice, *APO2*, is required to maintain IM indeterminacy and prevent a precocious transition to a terminal spikelet. (Ikeda-Kawakatsu et al. 2012). In *apo2* mutants, SAMs that usually aborts in wild-type (WT) are converted into terminal flowers because floral meristem identity acquisition occurs early. Further investigation into the function of APO2, particularly employing ChIP-seq, uncovered its numerous target genes (Miao et al. 2022). These include genes responsible for multiple stages of developmental transition, one of which could be the increase in meristem size, possibly shedding light on the underlying genetic machinery for the observed phyllotactic changes. In *Arabidopsis*, LFY and UFOs work together; their interaction enhances the ability of the resulting complex to recognize DNA sequences (Rieu et al. 2023) and cytoplasmic partitioning though condensate formation that marks LFY for degradation (Dolde et al. 2023).

### Plastochron

The plastochron changes as the plant enters its reproductive phase. In the vegetative phase, the plastochron pertains to the rate of leaf differentiation while in the reproductive phase, it equates to the differentiation rate of bracts and floral organs. In rice, there is a noticeable shortening of the plastochron during this transition. A leaf emerges roughly every 3–5 days during the vegetative phase (Miyoshi et al. 2004). However, in the reproductive phase, the formation of bract is theorized to occur even faster than this interval. More methods to quantitatively assess the bract formation rate in rice are needed. It is worth noting that in rice, mutants like *plastochron1 (pla1), pla2, pla3* and *apo1* and *apo2* result in a shortened plastochron during the vegetative phase (Miyoshi et al. 2004, Kawakatsu et al. 2006, 2009, Ikeda et al. 2007, Ikeda-Kawakatsu et al. 2012). However, whether these mutations impact the plastochron during the reproductive phase remains unclear. Investigating the regulation of the temporal intervals of organ differentiation promises to be an exciting avenue for future research.

Besides the acceleration of the plastochron, there is also acceleration in the timing of emergence of axillary bud meristem, considering the plastochron number of leaves and bracts when the axillary meristems emerge. In *Arabidopsis*, axillary meristems form from the axils of older leaves during the vegetative phase. However, after receiving flowering stimulus, this order is reversed and axillary mesirmtem development initiates from younger leaves (Long and Barton 2000). In rice, the meristems of the axillary buds that form during the vegetative phase are the meristems of the branched shoots that develop into vegetative shoots (Oikawa and Kyozuka 2009, Tanaka et al. 2015). This meristem is formed following these steps: at first, a premeristem zone is formed at P3 for generating the axillary meristem and then, the axillary meristem is first noticed at P4 in the axil. In contrast, a bract is a lateral organ during the reproductive phase, and primary branch meristems are formed in its axil (Ikeda et al. 2004). The axillary meristems emerge rapidly during the reproductive phase, appearing in two or three bracts ahead of the inflorescence meristem (IM) soon after its formation.

### Lateral organ identity

One of the most significant shifts during the developmental transition is the identity of the lateral organ derived from the SAM and the axillary meristem that forms in its axils (Kwiatkowska 2008). In the vegetative phase, lateral organ primordia derived from the SAM become leaves, and their axillary meristems become vegetative shoots. On the other hand, in the reproductive phase, these primordia take on the identity of bracts, a reduced leaf form. The axillary meristems of this phase become either branch meristems or floral meristems of the inflorescence (Ratcliffe et al. 1999, Kyozuka et al. 2014). Suppression of inflorescence leaf or bract is termed as ‘bract suppression’ and is observed in many angiosperms (Whipple et al. 2010). The genes that control bract suppression have been isolated and studied, revealing divergent regulatory systems between *Arabidopsis* and grasses (Xiao et al. 2022). In *Arabidopsis*, genes such as *PUCHI, UFO* and *LFY* play a crucial role in bract suppression (Karim et al. 2009). In contrast, the grass species uses a different set of genes, with GATA transcription factors such as *Neck Leaf1* (in rice), *Third Outer Glume* (in barley) and *Tassel Sheath* (in maize) being central (Wang et al. 2009, Whipple et al. 2010, Houston et al. 2012). An interesting observation in rice is that the *pla* mutants have dysfunctional bract repression, resulting in the development of bracts into vegetative mature leaves (Miyoshi et al. 2004, Kawakatsu et al. 2006, 2009, Wang et al. 2021). Genetic studies have identified a regulatory module that controls this process. Central to this process is the NL1 regulatory system, a key player in bract suppression. *NL1* expression is directly activated by SPL transcription factors, including OsSPL14, which is known to increase rice yield (Jiao et al. 2010, Miura et al. 2010). Furthermore, the accumulation of these SPL transcription factors is inhibited by miR156, whose levels decrease with plant age. Interestingly, NL1 cooperates with SPL transcription factors to increase *PLA1* expression, culminating in bract repression (Wang et al. 2021).

### Axillary meristem identity

In grass, the identity of axillary meristems in the axils of lateral organs is intrinsically linked to the presence or absence of bracts. For example, in rice *pla* and *spl* mutants, the bracts develop into leaves as in the vegetative phase (Miyoshi et al. 2004, Kawakatsu et al. 2006, 2009, Wang et al. 2021). At the same time, the identity of the axillary meristem shifts from the branched meristem of an inflorescence to a meristem of the vegetatively growing shoot (Miyoshi et al. 2004, Kawakatsu et al. 2006, 2009, Wang et al. 2021). The *MADS-box* genes play a role in this transition (Kobayashi et al. 2012). Specifically, in rice, *MADS-box* transcription factors from the FUL and SEP clades have been associated with a phenotype in which leaf differentiation continues after the developmental-phase transition (Kobayashi et al. 2012). The *OsMADS14, 15, 18* RNAi and *osmads34/pap2* quadruple loss-of-function rice plants show alterations in SAM doming and phyllotaxy, indicating an incomplete developmental phase transition (Kobayashi et al. 2012). Nevertheless, these plants still differentiate leaves, and their axillary buds are thought to be shoots. In wheat, several mutants of the genes encoding the MADS-box transcription factors in the FUL clade have shown failure in bract repression and development of a shoot within the inflorescence (Li et al. 2019, 2021). *VRN1*, a gene responsible for vernalization in wheat, encodes a MADS-box transcription factor in the FUL clade (Yan et al. 2003). Mutations in *vrn1* and its homologs, *ful2* and *ful3*, have shown this phenotype (Li et al. 2019). These findings are noteworthy because they underscore a link between bract identity and the specification of axillary meristem identity through phase transitions. Genetic analysis in maize suggests a model in which the bract produces indeterminacy and determinacy signals emanating from the suppressed bract (Xiao et al. 2022). Given that the *SPL* and *MADS-box* genes mentioned here are target genes of florigen in the SAM in many plants, these observations may pave the way for a more nuanced understanding of the developmental phase transition induced by the proteins encoded by *FT* or its orthologs that function as florigen. Importantly, TFL1, a protein structurally similar to FT, plays a critical role in establishing the identity of axillary meristems. TFL1 has a developmental function opposite to that of FT. In *tfl1* mutants, axillary meristem is transformed into floral meristem, highlighting the essential role of TFL1 in maintaining the shoot identity of axillary meristem (Conti and Bradley 2007).

## Understanding the Distribution and Function of Florigen in and around the Shoot Apical Meristem

### Transport of the proteins encoded by FT and its orthologs to the SAM

**Fig. 3:**
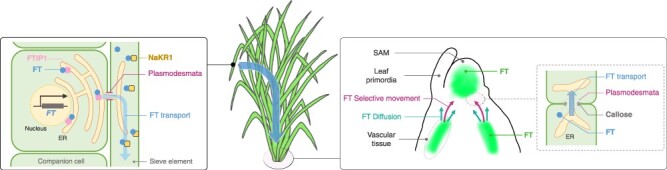
Transport and distribution of florigen FT. (Left) The FT gene is transcribed and translated within the phloem companion cell. Following this, the FT protein interacts with the FTIP1 protein located on the ER. Subsequently, the FT protein is transported to the sieve element through plasmodesmata. In the sieve element, FT interacts with the NaKR1 protein that facilitates transport through the phloem. (Right) Upon arrival to the shoot apex, FT is unloaded from the vascular tissue and directed toward the SAM, facilitated by a combination of selective movement and diffusion. The transport of FT is believed to be modulated by callose, a compound that regulates the permeability of plasmodesmata. Within the SAM, FT proteins do not extend to its periphery.

Understanding the vegetative-to-reproductive phase transitions requires thorough examinations of the distribution of the proteins encoded by *FT* and its orthologs that function as florigen within the SAM (**Fig. 3**) (Abe et al., 2019, Tamaki et al. 2015). Our knowledge in this area has significantly deepened due to advancements in our understanding of the florigen transport mechanisms by grafting and state-of-the-art imaging techniques (Notaguchi et al. 2008, Endo et al. 2018, Abe et al., 2019, Kinoshita et al. 2020, Sato et al. 2021, Sakamoto et al. 2022). The gene responsible for florigen *FT* in Arabidopsis and *Hd3a* in rice is predominantly expressed in leaf phloem companion cells (Takada and Goto 2003, Tamaki et al. 2007, Chen et al. 2018). From there, the FT and Hd3a protein is transported via the phloem to the SAM (Andrés and Coupland 2012, Tsuji 2017, Endo et al. 2018).

The transport of florigen is demonstrated by grafting experiments. Florigen is produced in phloem companion cells, where the *FT* undergoes transcription and translation before it is loaded into the phloem (**Fig. 3**) (Chen et al. 2018). Studies utilizing *FT* induction systems and grafting experiments in *Arabidopsis* have indicated that FT synthesized in leaves can reach the SAM in 12 h, demonstrating its efficient transportation dynamics (Notaguchi et al. 2008). FT-like proteins as florigen acts quantitatively through its transport, as shown by cabbage/radish inter-generic grafting (Motoki et al. 2022).

Several proteins responsible for the transport of FT or proteins encoded by *FT* orthologs have been identified and characterized. The C2 domain protein, FT-INTERACTING PROTEIN1 (FTIP1), plays a significant role in this intricate process in *Arabidopsis* (Liu et al. 2012). Despite the existence of multiple florigen genes in most plant species, it is suggested that each rice paralog may employ a distinct FTIP isoform (Song et al. 2017, Zhang et al. 2022). Two florigen proteins, Hd3a and RFT1, have been identified and characterized in rice (Komiya et al. 2008). Notably, Hd3a interacts with OsFTIP9, whereas RFT1 partners with OsFTIP1, a coupling essential to their function in transportation (Song et al. 2017, Zhang et al. 2022). SODIUM POTASSIUM ROOT DEFECTIVE 1 (NaKR1) in *Arabidopsis* is another regulator of FT transport. This mobile metal-binding protein may play a similar regulatory role (Zhu et al. 2016) and control *FT* expression through the availability of potassium nutrients (Negishi et al. 2018).

Another regulator of FT transport, recently identified, is lipid, which also acts as a modulator of FT function. In *Arabidopsis*, phosphatidylglycerol interacts with FT, impacting its transport (Susila et al. 2021). This interaction, reportedly stronger at lower temperatures, might inhibit FT translocation to sieve elements by anchoring it to the chloroplast membrane, a site of high phosphatidylglycerol concentration. Importantly, it has been observed that the phosphatidylcholine interacts with the FT and influences flowering in various species including *Arabidopsis*, rice, maize, and several *Rosaceae* plants (Nakamura et al. 2014, 2019, Wang et al. 2017, Qu et al. 2021, Barnes et al. 2022). These differences in lipid-binding specificity highlight the dynamic nature of this research area and suggest that future studies will provide further clarity on these interactions.

After transportation through the phloem, it is postulated that the proteins encoded by FT or its orthologs that function as florigen is unloaded at the vascular bundle terminus at the base of the SAM, from where it is transported toward the SAM (Yoo et al. 2013). Research on *Cucurbita moschata* suggests that diffusion and selective movement coincide after unloading, but selective movement becomes dominant as the protein product of *FT* orthologs in *C. moschata* approaches the SAM (**Fig. 3**) (Yoo et al. 2013). It is considered that three amino-acid substitutions on the surface of CmFT may inhibit this transport process (Yoo et al. 2013). Intriguingly, these amino-acid substitutions in *Arabidopsis* FT do not interfere with the interaction with FTIP1 (Endo et al. 2018). There may be an involvement of an as yet unidentified protein that interacts with FT in this transportation process.

Plasmodesmata-mediated intercellular communication is believed to play a crucial role in florigen transport (Yoo et al. 2013). In poplar, this mechanism is hypothesized to inhibit floral development signals by blocking cell-to-cell communications (**Fig. 3**) (Rinne et al. 2011, Tylewicz et al. 2018). Likewise, communication between the SAM cells by plasmodesmata is also proposed to be pivotal in lily bud dormancy (Pan et al. 2023).

### Distribution of the proteins encoded by FT or its orthologs that function as florigen in the SAM.

The distribution of florigen, once it reaches the SAM has been observed in two distinctive plant species—*Arabidopsis* and rice (**Fig. 3**) (Corbesier et al. 2007, Tamaki et al. 2007, 2015, Komiya et al. 2009, Abe et al. 2019). In *Arabidopsis*, FT-GFP expressed from phloem-specific *SUC2* promoter was detected within the SAM and provasculature before the emergence of the floral primordium (Corbesier et al. 2007, Liu et al. 2012); specifically, FT was found in a central region of the SAM, not at its periphery. This area largely coincides with where the FT–FD complex was observed within the SAM (Abe et al. 2019). These findings align with observations that Hd3a is not present at the periphery of the SAM in rice (Tamaki et al. 2007, 2015). In *Arabidopsis*, the distribution of FT has been estimated using an induction system in combination with bimolecular fluorescence complementation (BiFC) to visualize the FT–FD complex (Abe et al. 2019). The observation suggests that FT–FD complexes form within the floral anlagen, the initial primordial sites of floral organs. Interestingly, the localization of the FT–FD complex and the sites of expression of its crucial target gene, *AP1*, may not always be congruent. This suggests that the FT–FD complex might only transiently induce *AP1* expression or express FT functions via as-yet-unknown transcription factors. In contrast, the distribution of Hd3a in rice has been investigated by expressing Hd3a-GFP and RFT1-GFP under their promoters and directly observing the SAM (Tamaki et al. 2007, 2015, Komiya et al. 2009). Hd3a-GFP is notably absent in SAMs during the vegetative phase but becomes detectable during the reproductive phase. However, its distribution within the SAM is far from uniform and it tends to accumulate unevenly. Meanwhile, the expression of *OsMADS15*, a transcription factor gene in the *FUL* clade and a putative target of FT-like proteins in rice, shows a more widespread distribution than the Hd3a (Tamaki et al. 2015). These observations suggest that there may be an unidentified mechanism linking the restricted localization of Hd3a to the overall phase transition within the SAM. Deciphering the precise distribution of florigen within the SAM and unraveling the mechanism by which the phase transition signal emanates from the limited florigen distribution zone to engulf the entire SAM are important challenges that will steer the direction of forthcoming research in the field.

### Function and distribution of the proteins encoded by FT or its orthologs that function as florigen in tissues surrounding the SAM

Besides affecting the floral transition, the proteins encoded by FT or its orthologs also play a role in reproductive phases. This section outlines the multiple functions of these protiens, including seed dormancy, seed size, leaf form and internode elongation. In *Arabidopsis*, FT influences seed dormancy by regulating *FLOWRING LOCUS C (FLC)* transcription (Chiang et al. 2009, Chen and Penfield 2018, Luo et al. 2019). This FT-FLC modulation connects the parent plant’s environment with the dormancy of its seeds. The exact FT-FLC regulation mechanism is complex. Chen and Penfield showed that the mechanism involves transcriptional regulation of antisense RNA at the *FLC* locus (Chen and Penfield 2018). Luo et al. suggest that FT directly impacts the *FLC* promoter’s transcription (Luo et al. 2019). Rice FTL9 offers insights into how plants may measure a parent plant’s nutritional status to adjust seed morphology and quantity (Ta et al. 2023). While *FTL9* is a pseudogene in cultivated rice, *Oryza sativa*, it is functional in wild rice *Oryza rufipogon*. Genetic analysis in *O. rufipogon* shows the role of *OrFTL9* in balancing seed size and productivity by decreasing seed size but increasing seed quantity. Sugar stimuli activate *OrFTL9* expression in leaves during late reproductive phases when flowers develop. Both *OrFTL9* mRNA and protein are found in leaves, with the protein also detected in developing flowers, indicating the transportation of OrFTL9 from the leaves to the flowers. Thus, OrFTL9 likely operates as a long-distance developmental modulator, reflecting the parent plant’s photosynthetic efficiency, and determining the trade-off between flower size and number and optimizing its reproductive success based on environmental conditions (Ta et al. 2023). FT-like proteins control leaf morphology in various plants (Shalit et al. 2009, Tsuji et al. 2013b). In tomato, an *FT* ortholog *SINGLE FLOWER TRASS* (*SFT*) gene simplifies the compound leaf structure (Shalit et al. 2009). In rice, the final vegetative phase leaf, known as the flag leaf, has a distinct angle and shape (Tsuji et al. 2013a). Without florigen Hd3a and RFT1, rice plants cannot produce this leaf shape. The transcription factor OsFD2 aids flag leaf development by partnering with the rice florigen Hd3a. Hd3a is present in the flag leaf primordium. These findings hint at florigen being directed to the leaf primordium and altering leaf morphology. Additionally, florigen Hd3a may participate in the elongation of stem tissue located below the SAM (Nagai and Ashikari 2023). The repressor of stem elongation in rice, *DEC1/PINE1*, is expressed in the stem at the base of the SAM (Gómez-Ariza et al. 2019, Nagai et al. 2020) and its expression is suppressed during the developmental-phase transition. Thus, florigen might suppress the expression of *DEC1/PINE1* in the stem. Hd3a and FT are recognized as transcriptional co-activators. Nonetheless, their effect on *DEC1/PINE1* expression in rice is repressive. The precise molecular mechanism leading to this repression remains unclear. It may have a direct repressive effect in the chromatin context of Hd3a or it could be an indirect suppression linked to the developmental transitions induced by Hd3a.

## Molecular Dynamics during the Reproductive Phase Transition of SAM

Upon the arrival of florigen to cells within the SAM, it forms a transcriptional complex that orchestrates the reproductive phase transition (Taoka et al. 2013, Tsuji 2017). This is achieved through various molecular processes, notably activating specific target genes. The underlying mechanism propelling this transition has been the subject of extensive investigation from diverse angles. Three focus areas have received considerable attention:

1. The formation and function of the florigen activation complex.
2. The shifts in epigenetic regulation of transcription.
3. Reproductive phase transition as a trajectory of the transcriptional state of the SAM.

These areas of study have experienced significant progresses, bolstered by breakthroughs in genomics.

### The formation and function of the florigen activation complex

**Fig. 4:**
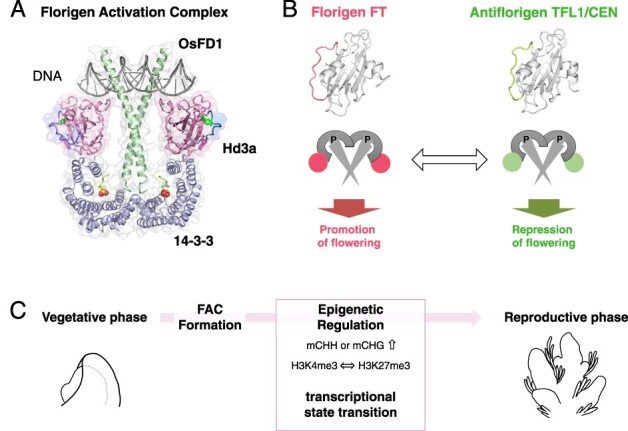
The FAC and the transition from vegetative-to-reproductive phase. (A) Structural model of FAC: the FAC comprises of the florigen FT, its receptor 14-3-3 and the bZIP transcription factor FD. The model is produced from the structure of rice homologs Hd3a, 14-3-3 and OsFD1. (B) Antagonistic functions at the molecular level: The florigen FT and antiflorigen TFL1/CEN operate in opposition. A conversion between FT and TFL1 shifts the function of the protein complex, toggling between promoting and repressing flowering. (C) Phase transition mediated by FAC: The formation of the FAC orchestrates the epigenetic regulation of gene expression. This subsequently catalyzes a shift in the transcriptional state of the SAM, leading to the vegetative-to-reproductive phase transition.

Florigen activation complex (FAC) is essential for the proper functioning of florigen in several plants (**Fig. 4A**) (Taoka et al. 2011, Li et al. 2015, Collani et al. 2019, Eshed and Lippman 2019, Liu et al. 2021). This complex comprises the florigen FT protein, the 14-3-3 protein and the FD transcription factor. Mutants with no interaction between Hd3a and 14-3-3 proteins exhibit complete loss of function of Hd3a in rice (Taoka et al. 2011). This shows that the function of rice Hd3a depends entirely on the formation of FAC. The cell biological analyses of rice suggest that FACs form through the following process: initially, Hd3a and 14-3-3 proteins interact in the cytoplasm of SAM cells, forming the Hd3a–14-3-3 subcomplex (Taoka et al. 2011). During this phase, Hd3a associates with 14-3-3, effectively concealing the 14-3-3 nuclear export signal (Taoka et al. 2013, Tsuji et al. 2013a). However, given that the size of the Hd3a–14-3-3 subcomplex surpasses those of the nuclear pore, the subcomplex remains localized within the cytoplasm. Upon expression of OsFD1 in the same cell, its C-terminal SAP motif undergoes phosphorylation (Abe et al. 2005, Taoka et al. 2011). The identification of a kinase that adds phosphate groups to FD has also been reported in *Arabidopsis* and rice (Kawamoto et al. 2015, Peng et al. 2021). The phosphorylated motif is subsequently recognized by the 14-3-3 protein, facilitating interactions between 14-3-3 and OsFD1. The subcellular localization and interaction of the three proteins were revealed in protoplasts. OsFD1 was mainly located in the nucleus. Hd3a-OsFD1 and GF14b-OsFD1 were both detected in the nucleus. In contrast, GF14b and GF14b-Hd3a were localized in cytoplasm and Hd3a in cytoplasm and nucleus. Co-expression of GF14b-Hd3a with OsFD1 resulted in the nuclear localization for all the components, and FRET-FLIM analysis showed the presence of three protein complex, the FAC, in the nucleus. FAC subsequently activates several downstream genes, including multiple *MADS-box* genes, which are crucial for the developmental phase transition in the SAM (Taoka et al. 2011). Intriguingly, in the rice SAM, Hd3a and RFT1 have been demonstrated to activate another *FT* gene, *OsFTL1*, which, when overexpressed, promotes floral development (Izawa et al. 2002). Transcriptome analysis reveals that *OsFTL1* is induced in the SAM early in the flowering process (Furutani et al. 2006) and regulates the inflorescence architecture (Wang et al. 2020). Furthermore, OsFTL1 enhances the effects of Hd3a and RFT1 in the transition from vegetative meristem to IM and subsequent panicle development (Giaume et al. 2023).

There is a large variation in the type and function of the transcription factor subunits of FAC. In *Arabidopsis*, FD and its paralog FDP vary in their function (Abe et al. 2005, Wigge et al. 2005, Romera-Branchat et al. 2020); both FD and FDP were thought to have promotive functions, but detailed analysis revealed that FDP is a transcription factor with repressive functions in flowering (Romera-Branchat et al. 2020). Rice also has several *FD* genes: *OsFD1, OsFD4* and *OsFD7* promote flowering, while OsFD2 regulates leaf development (Tsuji et al. 2013a, Cerise et al. 2021, Kaur et al. 2021). The possibility that AREB-type bZIP regulates flowering together with FT has also been noted in *Arabidopsis* (Martignago et al. 2023), and elucidating the FAC transcription factor repertoire will be an important topic of future genetic studies.

The discovery of FAC has broadened our understanding of florigen’s multifunctional nature and the repressive mechanism of antiflorigen TERMINAL FLOWER1 (TFL1)/CENTRORADIALIS (CEN) (**Fig. 4B**) (Taoka et al. 2013, Tsuji 2017). Here, we briefly describe the phosphatidylethanolamine-binding protein (PEBP) family to which florigen belongs. FT was first identified as a homolog of lipid-binding protein PEBP (Kardailsky et al. 1999, Kobayashi et al. 1999). PEBPs of plants can be classified into three major groups: a group of Mother of FT and TFL1 (MFTs) conserved from the basal plant, a group of florigen FTs, and an antiflorigen TFL1/CEN group (Bennett and Dixon 2021). MFT is considered the ancestral form and regulates seed germination (Xi et al. 2010, Nakamura et al. 2011, Yoshida et al. 2022, Lu et al. 2023). While MFT was previously considered incapable of interacting with 14-3-3 due to the lack of amino-acid residues essential for interaction for Hd3a with 14-3-3 (Taoka et al. 2011), recent reports suggest that OsMFT2 activates germination by interacting with the epsilon-type 14-3-3, which cannot interact with florigen FT (Yoshida et al. 2022). FT and TFL1/CEN exhibit a high level of structural similarity and share conserved interaction regions with 14-3-3 (**Fig. 4B**). However, they differ in the specific amino-acid residues on the protein surface, the surface charge and structure of segment B, a loop region that protrudes outside the protein and does not assume a structure (Ahn et al. 2006, Ho and Weigel 2014). The specific amino-acid mutations, such as FT W138E and TFL1 H88Y, that change FT activity to TFL1-like inhibitory function and vice versa (Hanzawa et al. 2005, Ho and Weigel 2014). As a result, they have opposite functions in promoting floral development. Specifically, FT promotes flowering, whereas TFL1 inhibits it. The mechanism for TFL1 suppression of flowering has long been unclear, but the discovery of FAC has improved our understanding of its central role (Kaneko-Suzuki et al. 2018). The rice TFL1 homolog RICE CENTRORADIALIS (RCN) loses its function when it loses the binding ability to 14-3-3, suggesting the critical role of 14-3-3-mediated complex formation in the functional expression of antiflorigen. The localization of antiflorigen in the SAM has been clarified in some instances, indicating an overlapping distribution pattern with florigen FT in *Arabidopsis* and rice (Conti and Bradley 2007, Kaneko-Suzuki et al. 2018). *TFL1* mRNA accumulates in the central region of the SAM in *Arabidopsis*. It is from this location that the TFL1 protein diffuses throughout the SAM, indicating that antiflorigen in *Arabidopsis* is regulated in the SAM (Conti and Bradley 2007). TFL1 distribution within the SAM has been extensively studied, with findings from Goretti et al. (2020) showing its pervasive presence throughout the SAM, predominantly accumulating in the central regions but also reaching the L1 (Goretti et al. 2020). Additionally, TFL1 accumulation has been observed in axillary meristems (Zhu et al. 2020). Genome-wide chromatin association analysis by ChIP-seq has revealed that TFL1 is recruited to the chromatin by FD (Zhu et al. 2020). Notably, in the *fd* mutant, the association of TFL1 with chromatin is almost completely lost (Zhu et al. 2020). Furthermore, TFL1 and FD are directly bound to 604 loci which are derepressed following a shift to inductive photoperiods in an FT-dependent manner. A key discovery in this context is that FT can displace TFL1 from the chromatin at direct target sites, which regulates vegetative to IM (V to I1 transition) affecting *CO* and *GI*, and from IM stage 1 to stage 2 (I1 to I2 transition) impacting genes like *LFY, AP1, FUL and LMI2.* These insights provide a deeper understanding of the dynamic interplay between TFL1, FD and FT in regulating plant development and floral transition (Zhu et al. 2020).

In contrast to *Arabidopsis, RCN* in rice is transcribed just below the SAM, and its protein is transported a short distance to the SAM, indicating that antiflorigen in rice is a repressor transported into the SAM from outside (Kaneko-Suzuki et al. 2018). These two proteins have different regulatory distributions, suggesting a complex regulation of their antiflorigen molecular function. A model has been proposed where the relative levels of FT and TFL1 regulate floral behavior in *Arabidopsis* (Jaeger et al. 2013). This model consolidates gene expression in the SAM into four central hubs: FT, TFL1, LFY and AP1. It highlights that the upregulation of TFL1 in response to FT signaling is pivotal in maintaining undifferentiated cells within indeterminate inflorescences.

In addition to advancing our understanding for antiflorigen TFL1/CEN, FAC is also involved in other functions of FT-like proteins, which extend beyond the induction of flowering. These functions include promoting the formation of potato tubers and rice branching (Tsuji et al. 2015, Teo et al. 2017). In addition to genetic validation, research using compounds targeting FAC formation reveals a range of florigen functions reliant on FAC assembly (Taoka et al. 2022).

The transcription factor FD is a pivotal anchor, holding FAC to DNA (Taoka et al. 2011). Advances in genome-wide analyses of the DNA-binding properties have significantly deepened our understanding of the regulatory mechanisms underlying FAC-DNA interactions. In *Arabidopsis*, a study using chromatin immunoprecipitation sequencing (ChIP-seq) of FD revealed that these binding sites (Goretti et al. 2020, Romera-Branchat et al. 2020, Zhu et al. 2020). Reexamination of the datasets reveals a significant consistency in FD-binding sites across different developmental stages, photoperiodic growth conditions and genetic backgrounds (Zhu et al. 2021). The critical role of FD in facilitating the interaction of FT and TFL1 with DNA is highlighted by the observed co-localization of TFL1 and FD at identical genomic loci (Zhu et al. 2020). This suggests that FD acts as a pivotal anchor, enabling TFL1 to associate with specific regions of the chromatin. Importantly, the presence of TFL1 at these chromatin sites is heavily dependent on the presence of FD, indicating a direct interaction between these proteins in the regulation of gene expression. Further emphasizing the complex regulatory network in floral development, LFY, a key regulator of floral fate, is found to be regulated in opposing ways by TFL1 and FT. Additionally, ChIP-qPCR analyses of other genes have shown that FT and TFL1 are in competition at shared loci (Zhu et al. 2020). It is reported that the association of FD to the promoter regions of numerous genes are modulated by the presence or absence of FT (Collani et al. 2019). This report proposes that one potential function of FAC assembly is to modulate the DNA-binding specificity of the complex.

The binding motifs of FD have been studied in parallel in rice and maize, revealing intricate regulatory mechanisms (Sun et al. 2020, Cerise et al. 2021). In maize, ChIP-seq analysis of DLF1, an FD homolog, has shown its affinity to promoters of several pivotal *MADS-box* genes, emphasizing the conservation of function across species (Sun et al. 2020). DNA affinity purification sequencing (DAP-seq), a method dedicated to profiling protein–DNA interactions *in vitro* (O’Malley et al. 2016), highlighted OsFD1’s binding affinity to the promoters of crucial *MADS-box* genes in rice (Cerise et al. 2021). Nevertheless, it is intriguing that not all *MADS-box* genes within the *AP1/FUL* subgroup exhibit OsFD1 binding, even though they are generally considered to be potential OsFD1 targets (Cerise et al. 2021). These findings imply that the landscape of FAC-mediated gene regulation is more complex than presently understood. Therefore, obtaining a comprehensive understanding remains an essential objective in the field.

### The shifts in epigenetic regulation of transcription

DNA methylation patterns in SAMs have been extensively studied in both rice and *Arabidopsis* (Nguyen and Gutzat 2022). DNA methylation occurs in the CG, CHG and CHH contexts where H represents the nucleotide residue other than C. Intriguingly, rice exhibits a genome-wide elevation in CHH methylation upon vegetative–reproductive phase transition (**Fig. 4C**) (Higo et al. 2020). One potential functional implication of this increase may be the suppression of transposable elements (TEs) in the SAM, followed by germ cell development in the subsequent reproductive growth. Notably, during the phase transition of SAMs, elevated CHH methylation is prominently associated with a class of TEs known as miniature inverted-repeat transposable elements (MITEs). The increased methylation could reinforce the silencing of MITEs. Notably, about half of the MITEs with methylation alterations display changes in germ cells. A previous study showed that germ cells undergo genome-wide DNA methylation reprogramming during differentiation, ensuring a dynamic epigenetic landscape (Yvonne Kim et al. 2019). The exact timing of the onset of these changes is yet to be determined, but the data suggest that methylation modifications of a proportion of MITEs begin during the developmental phase transition of the SAM. In this study, the transcriptome of small RNAs and the proteome analyses were conducted using purely isolated SAMs. An integrated analysis of these data, alongside the SAM transcriptome results, suggests that post-transcriptional activation of the RNA-directed DNA methylation (RdDM) pathway may be the mechanism driving CHH methylation (Higo et al. 2020). In *Arabidopsis*, the focus was on the stem cell region of the SAM. DNA methylome analysis was performed using a reporter strain with *CLV3* expression localized to the stem cell region at the SAM apex (Gutzat et al. 2020). Cells from this region were enriched using fluorescence-activated cell sorting (FACS), revealing a notable increase in methylation. Within stem cells of *Arabidopsis*, CHG methylation within TEs increases continuously, while CHH methylation decreases during flowering (**Fig. 4C**). These findings highlight both similarities and differences between rice and *Arabidopsis*. More precisely, *Arabidopsis* shows differences in CHG methylation, while rice displays an increase in CHH methylation. In any case, both species suggest a shared possibility: early epigenetic reprogramming may play a role in protecting the genome within stem cells during germ cell development.

Chemical modifications of histone tails play a pivotal role in epigenetic regulation (Ueda and Seki 2020, Ono and Kinoshita 2021). These modifications have been studied in the SAM of *Arabidopsis* and rice (Nguyen and Gutzat 2022). A modified version of the INTACT method designed for SAM was used to purify nuclei from cells in *Arabidopsis* (You et al. 2017). The study focused on two histone marks: H3K4me3, typically associated with active transcription of genes, and H3K27me3, which often corresponds to transcriptional repression. Findings indicated that H3K4me3 is predominantly linked with the transcriptional activation processes in flowering. On the other hand, the association of H3K27me3 with transcriptional repression is not unequivocal. Notably, two distinct histone modification states were identified in the *Arabidopsis* SAM: the ‘harboring’ state (H-state) and the ‘embedded’ state (E-state). The H-state is marked by an extensive presence of H3K27me3 modifications with a relatively narrow peak of H3K4me3 modification observed within this region. In contrast, the E-state is characterized by a narrow peak of H3K27me3 in regions where H3K4me3 modifications are widespread. The E-state, a rarity, is particularly evident in the SAM and is predominantly associated with downregulated genes during the early stages of flowering. Furthermore, certain regions of the genome exhibit potential bivalent modifications, where markers of both transcriptional activation (H3K4me3) and repression (H3K27me3) coexist. Genome-wide investigations into H3K4me3 and H3K27me3 have been undertaken in rice (Liu et al. 2015). In light of the nonavailability of data derived from pure meristem isolates, one might cautiously infer that the existing data are more representative of the shoot apex and inflorescence. An interesting observation is that CKX2, a determinant of rice yield (Ashikari et al. 2005, Gupta et al. 2023), is modulated by H3K27me3. Furthermore, regions suggestive of bivalent modifications were identified, though these might seem bivalent due to the mixed nature of cell populations (Liu et al. 2015). In both maize and Arabidopsis, enhancers are marked by the simultaneous presence of H3K4me3 and H3K27me3 in mixed cell populations (Lu et al. 2019, Ricci et al. 2019). Development of techniques that enable a detailed analysis of SAM would be beneficial in the future. This could involve a sequential examination of multiple modifications and advanced spatial analysis techniques, particularly at the single-cell level. Additionally, it will provide deeper insights into recently investigated regulatory mechanisms, such as chromatin accessibility (Sijacic et al. 2018) and chromatin 3D organization (Zhang and Wang 2021), during the developmental phase transition of the SAM.

### Reproductive phase transition as a trajectory of the transcriptional state of the SAM

Epigenetic changes during reproductive phase transitions correlate with transcriptional state shifts, with florigen playing a pivotal role in influencing the transcriptome. By studying transcriptome dynamics, we can understand developmental transitions in plants (Izawa et al. 2011, Nagano et al. 2012, 2019, Loudya et al. 2021). With regards to the reproductive phase transition in the SAM, early studies also focused on transcriptome analysis at later stages of inflorescence development (Schmid et al. 2003, Furutani et al. 2006). The primary purpose was to identify important genes that regulate this process for further functional analysis. These studies have identified genes that may be markers in flowering and subsequent floral development. The transcriptome dynamics in the *Arabidopsis* SAM was analyzed by laser microdissection and showed the correlation of the gene expression with the floral commitment, a process that maintain the floral transition even if returned to non-inductive condition (Bradley et al. 1997, Torti et al. 2012). Recently, efforts to identify florigen target genes have intensified using various techniques. In *Arabidopsis*, a comprehensive analysis revealed that FT and TFL1 directly target and regulate 604 genes crucial for the transition from vegetative to I1 and I1 to I2 stages of floral development in *Arabidopsis* (Zhu et al. 2020). The *Hd3a*-induced expression system in rice SAM identified 15 highly expressed genes as a core set of florigen target genes (Mineri et al. 2023).

The analysis of transcriptome transitions as developmental phase transitions in SAM can be regarded as a novel area that expands upon prior studies. The tomato SAM study primarily drives this research (Park et al. 2012, Meir et al. 2021). Tomato SAM is 10 times bigger than rice, and it is easy to isolate pure tomato SAMs (Nakayama et al. 2023). This ease of access has facilitated a number of experiments. The transcriptome data analysis from tomato SAMs isolated at different developmental stages revealed a progressive change in transcription patterns during the growth phase transition. The association of the transcription pattern with morphological changes allowed us to identify a set of marker genes that accurately reflect the stages of morphological development. The expression of these genes revealed the specific transcriptional states where growth arrests occur in mutants with arrested development (Park et al. 2012). This approach offers a novel insight into phase transitions. Research on tomatoes was further refined to distinguish developmental phase transitions from morphological changes. The aim was to capture the changes in the SAM that occurred in less than one day when the morphological changes were less prominent (Meir et al. 2021). The study used the following procedure: several SAMs were isolated simultaneously, their transcriptomes were measured, and the developmental stages of the SAMs were determined based on the similarity of their transcriptomes. This method clarified that five transcriptional states are involved in developmental phase transition in SAMs undergoing the process. Notably, mutations in florigen *SFT* do not impact the transcriptional state directly, but alter the temporal progression of the process, instead. In simple terms, florigen function controls the timing of the developmental phase transition. Furthermore, the analysis uncovered a developmental phase transition mechanism that does not depend on florigen, highlighting the future potential of this analysis (Meir et al. 2021). The transcriptional changes, prior to morphological changes, is shown in Arabidopsis. Single far-red-enriched long-day photoperiod enabled to enhance photoperiodic FT induction (Hempel et al. 1997) and precisely define transcriptional changes occurring in response to this shift (Zhu et al. 2020).

Research on meristem trajectory has been extended to study axillary buds, which are critical for rice yield in rice (Takai et al. 2023, Yang et al. 2023). In particular, the outgrowth of axillary buds can increase the number of rice panicles, increasing yield. A fundamental discovery was the gene called *MORE PANICLES3 (MP3)*, which increases the number of ears and overall yield, especially when the concentration of CO_2_ around is high. The MP3 gene is a novel allele of *FINE CULM1(FC1)/OsTb1*, which encodes the TCP family transcription factors that suppress axillary bud outgrowth. To understand the function of the *MP3* allele of *FC1*, the axillary buds were sampled and their transcriptomes analyzed. The study showed that rice plants with the *MP3* allele exhibited accelerated development of their axillary meristems, leading to higher spikelet production and greater yields. The relationship between axillary bud transcriptomes and TCP transcription factors is well established in *Arabidopsis* (González-Grandío et al. 2013). Studies involving transcriptional profiling of axillary buds from wild-type and *branched1* (*brc1*) mutant plants, particularly under simulated shade conditions, have identified groups of genes with mRNA levels that are influenced by *BRC1* encoding a TCP transcription factor. Among these genes, there is a subset that shows increased expression levels of abscisic acid response genes. Additionally, two distinct networks of genes related to the cell cycle and ribosomes were found to be downregulated in these conditions (González-Grandío et al. 2013).

The method of capturing developmental transitions as changes in transcriptional status has also been used to compare developmental stages between plant species with distinct morphologies. An excellent example is a comparative study of maize and sorghum inflorescence development where transcriptional profiles were collected throughout inflorescence maturation on samples from both plant species (Leiboff and Hake 2019). The inflorescence structure of these two species shares a partial similarity, but exhibit significant differences (Takanashi 2023). The gene expression in maize and sorghum inflorescences was reconstructed as trajectories (Leiboff and Hake 2019). As a result, five maize tassels and four sorghum inflorescence developmental stages were identified and compared. For these stages, known genetic master regulators in maize and their syntenic orthologs in sorghum were analyzed. The relative timing of gene expression suggested that heterochronic gene regulation may be involved in sorghum inflorescence development, leading to sustained tissue growth, but not in maize (Leiboff and Hake 2019). The novel approaches explained in this section can be further facilitated by combination with multiple omics approach and deep phenotyping of plants (Hirayama and Mochida 2023).

## Conclusion

This review has extensively covered the intricacies of the developmental phase transition influenced by the proteins encoded by *FT* or its orthologs that function as florigen in the SAM. Our discussions have centered on three primary questions:

1. How does the distribution of florigen in the SAM change?
2. What molecular roles does florigen assume within the SAM cells, and how do these roles impact the state transition of the SAM?
3. How can we connect the florigen function with developmental changes in the SAM?

Advanced imaging techniques targeting SAMs are shedding light on florigen distribution. As we look ahead, future research aims to provide detailed observations at 3D, single-cell level, and promises to uncover the intricate interactions with regulators such as plant hormones and transcription factors. Regarding the function of florigen and transitions of the SAM at the molecular level, there is an anticipation to gather deeper insights into the epigenetic transcriptional regulation driven by FAC and transitions within the SAM, encompassing plants in natural settings. Considering the shifts in SAMs associated with developmental phase transitions, a promising avenue lies in bridging studies on developmental phase transitions with those focusing on changes in plastochron, phyllotaxis and identity shifts in lateral organs and axillary meristems. Deepening our insights into these areas will undoubtedly refine our understanding of the fascinating florigen-induced developmental phase transition in the SAM.

## Data Availability

No new datasets were generated or analyzed in this study.
